# Evaluation of Salivary and Serum Antioxidant and Oxidative Stress Statuses in Patients with Chronic Periodontitis: A Case-Control Study

**DOI:** 10.3389/fphys.2017.00189

**Published:** 2017-03-31

**Authors:** Fatemeh Ahmadi-Motamayel, Mohammad T. Goodarzi, Zohreh Jamshidi, Reza Kebriaei

**Affiliations:** ^1^Department of Oral Medicine, Dental Research Center and Research Center for Molecular Medicine, Hamadan University of Medical SciencesHamadan, Iran; ^2^Research Center for Molecular Medicine, Hamadan University of Medical SciencesHamadan, Iran; ^3^Department of Oral Medicine, Hamadan University of Medical SciencesHamadan, Iran; ^4^Hamadan University of Medical SciencesHamadan, Iran

**Keywords:** periodontitis, total antioxidant capacity, malondialdehyde, saliva, serum, oxidative stress, antioxidants, oral disease

## Abstract

**Aim:** Local bacteria stimulate polymorphonuclear neutrophils to release reactive oxygen species in periodontitis. Increased levels of oxidative stress play a significant role in the pathogenesis of periodontitis. Therefore, this study aimed to evaluate total salivary and serum antioxidant capacity and malondialdehyde in patients with chronic periodontitis.

**Materials and methods:** Fifty-five healthy subjects and 55 patients with chronic periodontitis, with an age range of 30–50 years, were evaluated. After clinical examination and case selection, unstimulated whole saliva was collected in the morning. Blood samples were taken from the antecubital vein. Total antioxidant capacity and malondialdehyde levels were evaluated by spectrophotometric assay. Data were analyzed with *t*-test, using Stata.11 software program.

**Results:** The periodontitis group exhibited lower salivary (0.16) and serum (0.36) total antioxidant capacity (*P* = 0.11) compared to the control group. Mean salivary malondialdehyde levels in the case and control groups were 0.80 ± 0.09 and 0.42 ± 0.08, respectively. The results showed significantly higher levels of salivary and serum malondialdehyde in the periodontitis group. Gender did not have any effect on antioxidant and oxidative stress levels.

**Conclusion:** This study indicated increased levels of salivary and serum oxidative stresses in patients with chronic periodontitis. Total antioxidant capacity was mildly lower in the saliva and serum of these patients. Higher malondialdehyde levels with no changes in antioxidant status can result in systemic and local complications in these patients.

## Introduction

Subgingival plaques in periodontitis have a plethora of microbial flora which cause inflammation and destruction of gingival tissues. Host cells release proinflammatory cytokines (interlukins 1, 6 and 8, and TNF-a) which stimulate infiltration of polymorphonuclear cells, the first line of cellular host defenses, against potential pathogens in the gingival sulcus. Polymorphonuclear cells produce proteolytic enzymes and O^2^ by oxidative burst. The interaction between bacteria increases oxygen consumption and produces reactive oxygen species (ROS) for killing of the bacteria which are capable of initiating periodontal tissue destruction (Sheikhi et al., 2001; Guentsch et al., 2008; Pendyala et al., 2008; Abou Sulaiman and Shehadeh, 2010; Žilinskas et al., 2011).

ROS is produced as a result of normal cellular metabolism processes (Akalin et al., 2007). It is important in cell signaling and metabolic processes (Brock et al., 2004; Gümüş et al., 2009).

Antioxidants (AOs) are substances that significantly delay or prevent oxidation of substrates that can be oxidized (Brock et al., 2004). AOs protect human body's cells from harmful oxidant effects. AOs eliminate oxidants and repair damage produced by ROS (Sheikhi et al., 2001). AOs are classified as chain- breaking AO, preventive AO and enzymes which control ROS (Baltacioglu et al., 2006; Akalin et al., 2007). AOs remove ROS which is necessary for safe aerobic life (Baltacioglu et al., 2006).

An imbalance between ROS production and antioxidant defense ability leads to oxidative stresses in tissues. ROS and oxidative stress have a main role in the pathogenesis of more than 100 inflammatory disorders (Brock et al., 2004; Gümüş et al., 2009; Tóthová et al., 2015) like periodontitis, diabetes, rheumatoid arthritis, stroke, and inflammatory lung diseases (Baltacioglu et al., 2006; Akalin et al., 2007; Chapple et al., 2007; Trivedi et al., 2015; Zhang et al., 2016).

ROS destroys DNA, lipid, protein, enzymes, and tissues (Sheikhi et al., 2001).

One of the most important reactions of free radicals is lipid peroxidation which changes the structural integrity and function of cell membranes. Malondialdehyde (MDA) is the end products of lipid peroxidation, as an oxidative stress marker, and results in tissue destruction by oxidative stress (Guentsch et al., 2008).

AO activities in saliva are strongly associated with periodontitis and various clinical variables (Tamaki et al., 2015). Several studies have shown relationships between periodontitis and oxidative stress and various clinical variables by measuring total antioxidant capacity (TAC) and MDA (Almerich-Silla et al., 2015; Baser et al., 2015; Tamaki et al., 2015; Trivedi et al., 2015). Despite contradictory results, some studies have shown a positive relationship between oxidative stress and periodontitis (Chapple et al., 2002, 2007; Brock et al., 2004; Baltacioglu et al., 2006; Akalin et al., 2007, 2009; Canakci et al., 2007, 2009; Guentsch et al., 2008; Pendyala et al., 2008; Žilinskas et al., 2011).

Recent data suggest increased oxidative stresses and MDA in periodontitis (Almerich-Silla et al., 2015; Trivedi et al., 2015; Zhang et al., 2016).

The hypothesis of this study was as follows: Periodontitis patients exhibit changes in local and systemic antioxidant and oxidative stresses. Saliva as a mirror of body health may reflect general health and may be used as a diagnostic material in future.

To the best of our knowledge, only one study to date has evaluated both salivary and serum antioxidant and oxidative stress statuses in periodontitis patients (Baltacioglu et al., 2014).

Given the controversies in the results of different studies and the importance of AO and oxidative stress levels in prevention, initiation, progression, and treatment of periodontal diseases, this study was undertaken to evaluate salivary and serum levels of TAC and MDA in chronic periodontitis in comparison to a healthy control group.

## Materials and methods

In this case-control study 110 subjects were selected. The sample size was calculated according to previous study results (Guentsch et al., 2008). Fifty-five healthy subjects and 55 patients with chronic periodontitis with an age range of 30–50 years were included. Each group consisted of 27 females and 28 males. All the subjects were informed of the study procedures and written informed consent was obtained from all of them. The study protocol was approved by the Ethics Committee of Hamadan University of Medical Sciences. The research was conducted in full accordance with the ethical principles of the World Medical Association Declaration of Helsinki. Subjects with systemic diseases and those taking medications, pregnant women, smokers, alcohol users, and addicted individuals were excluded.

The subjects in the case group were selected from patients referred to the dental clinics in Hamadan and Tehran for treatment of periodontal diseases. The control group subjects were selected from those referring for routine dental examinations with eligible criteria in Hamadan Dental School during 2010–2013. We matched the case and control groups in relation to age and gender. All the participants were 30–50 years of age.

Periodontal disease and its severity were defined in terms of probing depth and clinical attachment loss. A full-mouth periodontal examination was performed for clinical attachment loss. The distance between the CEJ and the bottom of the gingival crevice was defined as the clinical attachment loss.

Patients with moderate periodontitis had 3–4 mm of attachment loss and patients with severe periodontitis had attachment loss >5 mm. Periodontitis was defined as the presence of proximal and mesial to distal clinical attachment loss of >4 mm in two or more teeth (Page and Eke, 2007). We selected patients with moderate and severe periodontitis.

Unstimulated whole salivary samples were collected using sterile Falcon tubes in 5 min in the morning (Navazesh, 1993). The salivary sample were immediately placed on to ice and stored at 4°C and transferred to the laboratory in a maximum of 20 min and kept at –80°C until the analysis.

Blood samples from antecubital vein were taken between 8:00 and 9:30 a.m. The blood samples were centrifuged for 10 min at 3,000 rpm within 30 min after venipuncture.

MDA levels were measures on the basis of reaction with thiobarbituric acid. In this method MDA was mixed thiobarbituric acid and colorful pigments were produced that extraction of colorful material in excellent phase, intensity of fluorescent was measured with stimulation wavelength of 520 nm and issuance wavelength of 550 nm with the unit of nmol/mL (Rai et al., 2006).

TAC was measured by FRAP method. In this method the revival power of ferrous ion was measured in the presence of tripyridyltriazine and by formation of colorful complex. Ferrous -tripyridyltriazine and examination of the variation of light absorption in 593 nm with mol/mL unit (Riviere and Papagiannoulis, 1987).

Student's *t*-test and chi-squared test were used for data analysis, using Stata 11. Statistical significance was set at *P* < 0.05.

## Results

Table 1 shows gender characteristics of the case and control groups.

**Table 1:** **Characteristic of case and control group**.

**Sex**	**Number**	**Percent**	**Cum**.
Male	56	50.45	50.45
Female	55	49.55	100
Total	111	100	
**Sex**	**Health**	**Perio**	**Total**
Male	28	28	56
Female	28	27	55
Total	56	55	111

Mean salivary TAC levels in the case and control groups were 0.16 ± 0.009 and 0.18 ± 0.1 (mol/mL), respectively.

Periodontitis group had lower salivary and serum TAC levels but the difference was not statistically significant. Mean and standard deviation of each data are presented in Table 2.

**Table 2:** **Mean and standard deviation of salivary and serum total antioxidant capacity (TAC) in case and control group**.

**variables**	**Salivary TAC**	***P*-value**	**Serum TAC**	***P*-value**
	**Mean ± *SD***		**Mean ± *SD***	
Male	0.17 ± 0.1	*p* = 0.5	0.37 ± 0.04	0.95
Female	0.17 ± 0.02		0.37 ± 0.02	
Periodontitis	0.16 ± 0.09	*p* = 0.11	0.36 ± 0.01	0.11
Control	0.18 ± 0.1		0.37 ± 0.05	

*T-test*.

Mean salivary MDA levels in the case and control groups were 0.80 ± 0.09 and 0.42 ± 0.08, respectively.

The results showed significantly higher levels of salivary and serum MDA in the periodontitis group compared to the healthy control group.

Table 3 presents data in detail.

**Table 3:** **Mean and standard deviation of salivary and serum malondialdehyde (MDA) in case and control group**.

**variables**	**Salivary MDA**	***P*-value**	**Serum MDA**	***P*-value**
	**Mean ± *SD***		**Mean ± *SD***	
Male	0.62 ± 0.2	*p* = 0.44	1.47 ± 0.31	0.66
Female	0.59 ± 0.2		1.49 ± 0.37	
Periodontitis	0.80 ± 0.09	*p* = 0.0001	1.76 ± 0.09	0.0001
Control	0.42 ± 0.08		1.15 ± 0.18	

Gender did not have any effect on antioxidant and oxidative stress levels. Comparison of male and female subjects showed no statistically significant differences in their TAC and MDA levels (Tables 1, 2).

## Discussion

Adult periodontitis is one of the most common chronic inflammatory diseases, in which microbial plaque causes periodontal ligament and bone destruction. Bacterial colonization, host immune response, and genetic predisposition are some of the main etiologic factors (Michalowicz et al., 2000; Kinane and Lappin, 2001; Sheikhi et al., 2001; Pihlstrom et al., 2005; Pussinen et al., 2007; Žilinskas et al., 2011).

High oxidative stress and low antioxidant capacity might have important roles in the etiopathogenesis of periodontitis (Tsai et al., 2005; Almerich-Silla et al., 2015; Baser et al., 2015; Tamaki et al., 2015; Trivedi et al., 2015).

We studied the salivary and serum levels of MDA and TAC in chronic periodontitis. The results showed significantly higher levels of salivary and serum MDA in the periodontitis group compared to the healthy control group. Salivary and serum TAC did not exhibit any statistically significant difference between the two groups although the case group had lower TAC levels compared to the healthy controls.

Based on the results of this study, periodontitis can also induce systemic oxidative stresses and alter serum MDA levels and vice versa.

Other studies have shown a reduction in both systemic and local antioxidant capacity and antioxidant concentration of gingival crevicular fluids in the periodontitis group as compared with the controls (Brock et al., 2004; Baltacioglu et al., 2006; Canakci et al., 2007; Guentsch et al., 2008).

Consistent with the results of this study, in a study by Panjamurthy MDA level was significantly higher in the periodontitis group (Panjamurthy et al., 2005). Synthesis of MDA might be due to a decrease in AO in destroyed in periodontal tissues.

Celec et al., too, showed high salivary MDA levels in periodontitis. No correlation was observed between salivary and serum MDA levels in their study. They concluded that local oxidative stress is a predisposing factor for MDA production in periodontitis (Celec et al., 2005).

Trivedi et al. showed significant MDA elevation and reductions in antioxidant enzymes in periodontitis patients. They reported a direct correlation between MDA levels and an inverse correlation of antioxidant enzymes with periodontitis (Trivedi et al., 2015).

Our results are consistent with studies demonstrating an increase in lipid peroxidation levels in serum, saliva, gingival crevicular fluid and gingiva in periodontitis (Sobaniec and Sobaniec-Lotowska, 2000; Sheikhi et al., 2001; Mashayekhi et al., 2005; Panjamurthy et al., 2005; Tsai et al., 2005).

A number of recent studies measured different salivary oxidative stress markers in periodontitis and demonstrated that MDA was a more specific biomarker of lipid peroxidation in periodontitis patients. We assessed MDA which is a specific marker than others (Takane et al., 2002; Sculley and Langley-Evans, 2003; Sugano et al., 2003; Halliwell and Whiteman, 2004; Panjamurthy et al., 2005).

Similar to our results, in other studies salivary and serum TAC levels were lower in periodontal diseases compared to the control group (Baser et al., 2015; Tamaki et al., 2015).

One study suggested lower plasma antioxidant capacity in severe periodontitis, especially in the aggressive form and concluded that antioxidants might predict tissue destruction (Baser et al., 2015).

Based on our findings TAC was slightly lower in the case group. Zhang et al. also showed lower salivary TAC levels in periodontal patients (Zhang et al., 2016). We evaluated TAC because assays of TAC are biological interactions between individual antioxidants while specific antioxidant analysis might provide misleading information, and some of them might be undiscovered or difficult to assay, expensive, and time-consuming than TAC assays (Maxwell et al., 2006).

In our study we collected whole unstimulated saliva because it has major salivary gland composition and contains some elements of gingival crevicular fluids, immune cells, and tissue metabolites. It also most closely reflects the intraoral condition (Sculley and Langley-Evans, 2003; Tsai et al., 2005; Gümüş et al., 2009).

Oxidative stress and AO also change in smoking, diabetes mellitus, pregnancy, and systemic diseases. Therefore, we excluded all the subjects with systemic diseases and smokers from our study (Nagler et al., 2000; Canakci et al., 2007; Chapple et al., 2007; Gümüş et al., 2009).

In the present study gender had no effect on TAC and MDA levels, although other studies have shown gender differences in their levels (Brock et al., 2004; Maxwell et al., 2006). One study similar to the present study demonstrated that sex did not have any effect on gingival crevicular fluid TAC levels (Brock et al., 2004).

Based on other studies successful periodontal therapy increased gingival crevicular fluid TAC levels (Chapple et al., 2007) and decreased MDA levels (Guentsch et al., 2008); therefore, periodontal therapy can be very useful for the patient. TAC was significantly higher after scaling in a study by Yang et al. (2014).

It is still unclear whether periodontal disease leads to oxidative stresses or they happen as a result of it (Panjamurthy et al., 2005; Baltacioglu et al., 2006). Local and systemic antioxidant prescription might be helpful in periodontitis patients in lowering oxidative stress levels and might have beneficial effects on patients' general health.

In this study we only compared periodontitis patients with a healthy group and did not evaluate the effect of periodontitis severity on TAC and MDA levels.

Our results confirmed our primary hypothesis that local and systemic oxidative stresses and antioxidants play a role in periodontitis. Oxidative stress is produced in periodontitis, aggravating periodontal tissue destruction. MDA levels increased significantly and TAC levels decreased in periodontitis patients. Higher TAC levels can reduce MDA levels.

It seems our results had internal and external validity due to standard sample selection and examination. Therefore, the results of this study can be generalized and probably show the status of periodontal patients in the entire world.

The limitations of the present study were as follows: In this study we did not evaluate AO and oxidative stresses in terms of the disease severity and therapy. Studies on the measurement of MDA levels in terms of disease severity and therapy might be helpful in early diagnosis and prevention of periodontitis. In this study one trained postgraduate oral medicine student examined all the patients. It was advisable that the patients could be examined by two observers and they findings could be calibrated and the concordance between the two periodontal examinations calculated. In this study only TAC was evaluated as it can show all the antioxidant activity together. Evaluation of the subjects' antioxidants is recommended in future studies because it is very helpful.

Recently there has been more interest is prevention of disease by specific nutrient antioxidants (Panjamurthy et al., 2005; Canakci et al., 2007); therefore, further longitudinal investigations on large study populations, with age- and sex-matched groups, different periodontal status, and salivary, serum, plasma, and gingival crevicular fluid TAC and MDA levels and individual antioxidants are recommended to understand the mechanisms involved and whether it is the cause or effect of the disease.

The data presented in the current study indicated that periodontitis group had increased oxidative stress levels than the healthy control group. TAC mildly compromises in the saliva and serum of these patients. This high salivary and serum MDA levels with no change in antioxidant status can cause systemic and local complications in these patients.

## Conclusion

This study indicated increased levels of salivary and serum oxidative stresses in patients with chronic periodontitis than a healthy control group. TAC was mildly lower in the saliva and serum of these patients. These high salivary and serum MDA levels, with no change in antioxidant status can cause systemic and local complications in these patients.

## Author contributions

FA Idea, conception, and design of the work; Data collection; Data interpretation; Drafting of article; Final approval of article. MG Sialochemichal Analysis; Data collection; Final approval of article. ZJ Data acquisition; Data analysis; Final approval of article. RK Data collection; Final approval of article. Agreement to be accountable for all aspects of the work in ensuring that questions related to the accuracy or integrity of any part of the work are appropriately investigated and resolved.

### Conflict of interest statement

The authors declare that the research was conducted in the absence of any commercial or financial relationships that could be construed as a potential conflict of interest.

## Abbreviations

ROS: Reactive oxygen species
AO: Antioxidants
MDA: Malondialdehyde
TAC: Total antioxidant capacity.
